# Supplementary material for: Characterization of SOD1-DT, a Divergent Long Non-Coding RNA in the Locus of the SOD1 Human Gene
Source: Cells. 2023 Aug 13;12(16):2058. doi: 10.3390/cells12162058 (PMC10453398; doi:10.3390/cells12162058)
Supplement: Supplementary file 1 [file cells-12-02058-s001.zip › cells-2505910-supplementary tables.pdf]

Table S1. Primary fibroblasts

ALS 1	p.D12Y	FALS	F
ALS 2	p.L68P	SALS	F
ALS 3	p.L85F	FALS	F
ALS 4	p.D91A	SALS	F
ALS 5	p.G94D	FALS	M
ALS 6	p.E133del	SALS	M
ALS 7	p.S135N	FALS	M
ALS 8	p.I150T	FALS	F
CTRL 1	-	HEALTHY	M
CTRL 2	-	HEALTHY	M

Table S2. DNA samples from ALS patients

1	p.D11Y	FALS	F
2	p.D11Y	FALS	F
3	p.L84F	FALS	F
4	p.L84F	FALS	M
5	p.L84F	FALS	M
6	p.L84F	FALS	M
7	p.D90A	FALS	M
8	p.G93D	FALS	M
9	p.I149T	FALS	F
10	p.I149T	FALS	M
11	p.L144F	FALS	M
12	p.L144F	FALS	M
13	p.L144F	FALS	M
14	p.L144F	FALS	M
15	p.A4V	FALS	F
16	p.S134G	FALS	M
17	p.L38R	FALS	M
18	p.S134N	FALS	M
19	p.D11Y	SALS	F
20	p.D11Y	SALS	M
21	p.D90A	SALS	F
22	p.D90A	SALS	F
23	p.D90A	SALS	M
24	p.D90A	SALS	F
25	p.D90A	SALS	M
26	p.D90A	SALS	F
27	p.D90A	SALS	F
28	p.G93D	SALS	F
29	p.G93D	SALS	M

30	p.G72S	SALS	M
31	p.F20L	SALS	F
32	p.G85S	SALS	M
33	p.V87M	SALS	M
34	p.N19S	SALS	F
35	p.G86S	SALS	M
36	p.L67P	SALS	F
37	p.E133del	SALS	M
38	p.H49N	SALS	F
39	p.A141D	SALS	F
40	Unknown	FALS	M
41	Unknown	FALS	F
42	Unknown	FALS	M
43	Unknown	FALS	F
44	Unknown	FALS	M
45	Unknown	SALS	M
46	Unknown	SALS	F
47	Unknown	SALS	M
48	Unknown	SALS	M
49	Unknown	SALS	M
50	Unknown	SALS	M
51	Unknown	SALS	M
52	Unknown	SALS	F
53	Unknown	SALS	F
54	Unknown	SALS	F
55	Unknown	SALS	F
56	Unknown	SALS	M
57	Unknown	SALS	F
58	Unknown	SALS	F
59	Unknown	SALS	M
60	Unknown	SALS	M
61	Unknown	SALS	F
62	Unknown	SALS	M
63	Unknown	SALS	M
64	Unknown	SALS	F
65	Unknown	SALS	M
66	Unknown	SALS	M
67	Unknown	SALS	M
68	Unknown	SALS	F
69	Unknown	SALS	M
70	Unknown	SALS	F
71	Unknown	SALS	F
72	Unknown	SALS	M

Table S3. Primers for PCR and qPCR

Gene	Sequence	Product length (bp)
SOD1-AS T1	TCCCTTTAAGGCACATAATTTTCTT	106
	AACCGCACAAACTTTAGGCA	
SOD1-AS T2-T3	TGCAGTACGCGAAATTGGCA	63-193
	CCCTTGTTCAGGTGTGACGA	
SOD1-AS T2-T3	TGCAGTACGCGAAATTGGCA	386-518
	CGGACACGCCACCTTTAAGA	
L34	GTCCCGAACCCCTGGTAATAGA	166
	GGCCCTGCTGACATGTTTCTT	
SOD1	GTGAAGGTGTGGGGAAGCAT	140
	TTTGGCCCACCGTGTTTTCT	
TIAM1	GGAGCAGGTTTTCTTCTCTGTGG	101
	TACAGCTTCGGTTCCCTCTCCA	
SCAF4	CTACTCAGCAGGATGTTGCCAG	144
	TTCCTCGGCTCAGTTTCTGCAG	
CFAP298	AGATGCCTTGGACCAGCTTCGA	143
	CTGCCTCTTTAATGACGTTGAGC	
MIS18A	CGGACACCAACTGCATCCTGCTT	144
	CGTAGCCAAGATTGAGTGAGCAC	
URB1	CTCGCTGTTTCTGCCGACTCTA	134
	CAAAGTGGCTGCTCTCACAGAC	
HUNK1	TCAACACTGTGCTCTCCAACCG	117
	TGTAGCAGAGGCTGTCCTGGAT	
CCT8	CTGTTGGTGCTACAGCTCTTCC	103
	ACCACCTGAGTATCTCCAACTTC	
FBXW11P1	ATGAGCGTGTCATTGTAACTGGC	129
	GTCCATTGCTGAAGCGTAAGTGC	

Table S4. TFs analysis with SCREEN

Factor	# of experiments that support TF binding	# experiments in total
POLR2A	69	82
CTCF	50	180
TAF1	11	11
YY1	10	13
MYC	9	10
