# Supplementary material for: Characterization of SOD1-DT, a Divergent Long Non-Coding RNA in the Locus of the SOD1 Human Gene
Source: Cells. 2023 Aug 13;12(16):2058. doi: 10.3390/cells12162058 (PMC10453398; doi:10.3390/cells12162058)
Supplement: Supplementary file 1 [file cells-12-02058-s001.zip › Suppl_figures_legends.pdf]

Suppl. Figure S1. SH-SY5Y cells differentiation upon treatment with retinoic acid (RA). (A) Experimental design of SH-SY5Y differentiation: cells were seeded and treated with 10 μM of RA for the indicated days. Undifferentiated (D0) and differentiated (D6) cells has been used for RNA extraction. Neurites length (μm) extending from SH-SY5Y during the differentiation was assessed. An immunofluorescence assay was also performed. Cells were stained with βIII-Tubulin antibody (green) and DAPI (blue). (B) QPCR of SOD1 in differentiated cells. Data are expressed as: mean ± SD, **** p value ≤ 0.0001; ns = not significative; test = One-way ANOVA for neurite length and paired t-test for qPCR analyses.

Suppl. Figure S2. Western Blot for YY1 and ACTIN proteins in SH-SY5Y cells.

Suppl. Figure S3. QPCR analysis of SCAF4, CFAP298, MIS18A and URB1 genes in SOD1-DT silenced cells compared to the control.
