# Supplementary figures and images for: Characterization of SOD1-DT, a Divergent Long Non-Coding RNA in the Locus of the SOD1 Human Gene
Source: Cells. 2023 Aug 13;12(16):2058. doi: 10.3390/cells12162058 (PMC10453398; doi:10.3390/cells12162058)

A

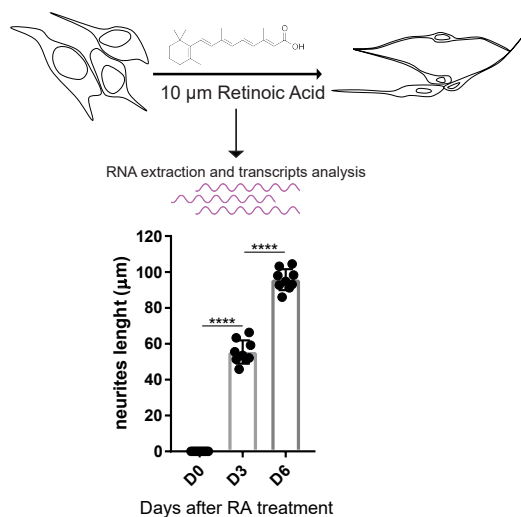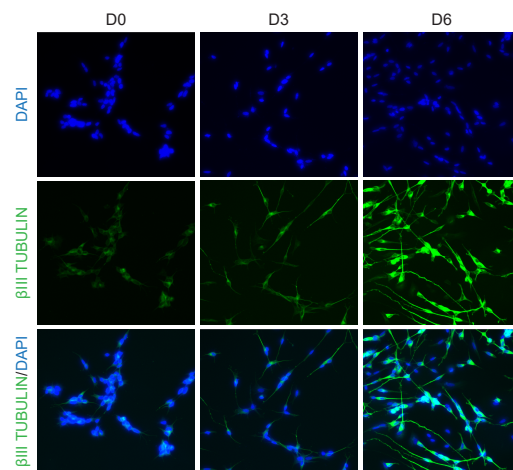

B

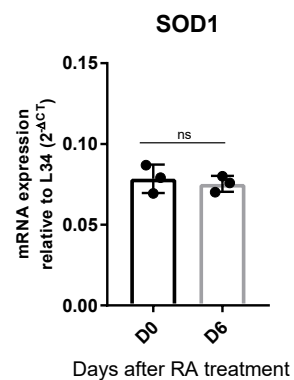

Suppl. Figure S1

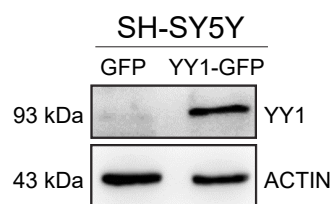

Suppl. Figure S2

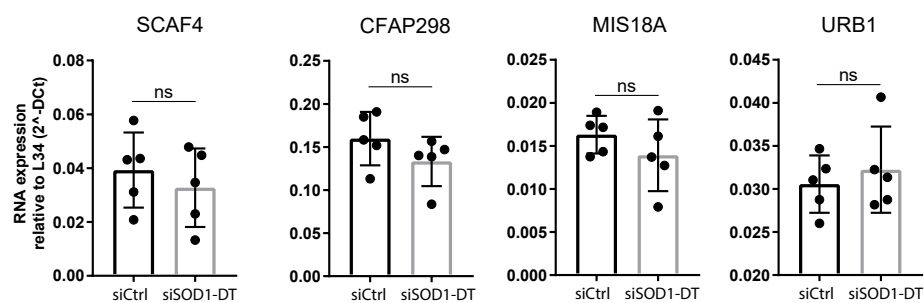

Suppl. Figure S3

Supplement: Supplementary file 1 [file cells-12-02058-s001.zip › Suppl.Figures.pdf]
